# The co-expression of cytokeratin and p63 in epithelioid angiosarcoma of the parotid gland: a diagnostic pitfall

**DOI:** 10.1186/1746-1596-7-118

**Published:** 2012-09-03

**Authors:** Xu-Yong Lin, Yang Liu, Yong Zhang, Juan-Han Yu, En-Hua Wang

**Affiliations:** 1 Department of Pathology, The First Affiliated Hospital and College of Basic Medical Sciences, China Medical University, Shenyang, 110001, China; 2 Institute of pathology and pathophysiology, China Medical University, Shenyang, 110001, China

**Keywords:** Angiosarcoma, Cytokeratin, p63

## Abstract

**Summary:**

Epithelioid angiosarcoma of the parotid gland is rare, and may pose a great diagnostic challenge. We report a case of primary epithelioid angiosarcoma in a 64-year-old male without history of radiation. The histopathological findings demonstrated a high grade epithelioid neoplasm. Immunostaining showed that the tumor was positive for the pan-cytokeratin, p63, cytokeratin18, Vimentin and vascular markers CD31, and was negative for CD34, cytokeratin5/6, cytokeratin7, cytokeratin20, CD68, CD30, S-100, HMB45, desmin, α–SMA and CD45. The tumor was diagnosed as an epithelioid angiosarcoma. To our knowledge, this is the first reported case of angiosarcoma which showed common positivity for cytokeratin and p63. In addition to cytokeratin, p63 is considered a useful marker for carcinoma. The co-expression of cytokeratin and p63 in epithelioid angiosarcoma represents a diagnostic pitfall. Thus, using a panel of antibodies is essential for distinguishing this tumor from poorly differentiated carcinoma.

**Virtual Slides:**

The virtual slide(s) for this article can be found here: http://www.diagnosticpathology.diagnomx.eu/vs/6548916707504750

## Background

Angiosarcoma predominantly arises in the skin or superficial soft tissue. The majority of neoplasms of the parotid gland are epithelial, while angiosarcoma, especially epithelioid angiosarcoma is extremely rare [1,2]. Because of its rarity, epitheloid angiosarcoma may be misdiagnosed as an epithelial tumor [3]. Angiosarcoma is a highly aggressive tumor. Local recurrences develop in about one fifth of patients and one half may be expected to die within the first year after diagnosis with metastatic disease in the lung followed by lymph node, bone, and soft tissue [4]. So, it is necessary to distinguish epithelioid angiosarcoma from poorly differentiated squamous cell carcinoma or other epithelioid lesions. Here, we report a case of primary epithelioid angiosarcoma of the parotid gland in a 64-year-old Chinese male without history of radiation to the head and neck.

## Case history

A 64-year-old male without a previous history of malignancy or radiation in the head and neck area presented with a painful swelling of the right parotid gland. The patient reported the parotid gland increased rapidly in size. A parotidectomy was then performed in our hospital. Three months later, the tumor recurred in the same site, and then the tumor was excised again.

## Methods

The resected specimens were fixed with 10% neutral-buffered formalin and embedded in paraffin blocks. Tissue blocks were cut into 4-μm slides, deparaffinized in xylene, rehydrated with graded alcohols, and immunostained with the following antibodies: pan-cytokeratin (pan-CK), cytokeratin 5/6 (CK 5/6), cytokeratin18 (CK18), p63, vimentin, CD31, CD34, cytokeratin7 (CK7), cytokeratin20 (CK20), S100, desmin, α–smooth muscle actin(α–SMA), CD45, CD30, CD68, HMB45 and Ki67. Sections were stained with a streptavidin-peroxidase system (KIT-9720, Ultrasensitive TM S-P, MaiXin, China). The chromogen used was diaminobenzidine tetrahydrochloride substrate (DAB kit, MaiXin, China), slightly counterstained with hematoxylin, dehydrated and mounted.

## Results

**Figure 1:**
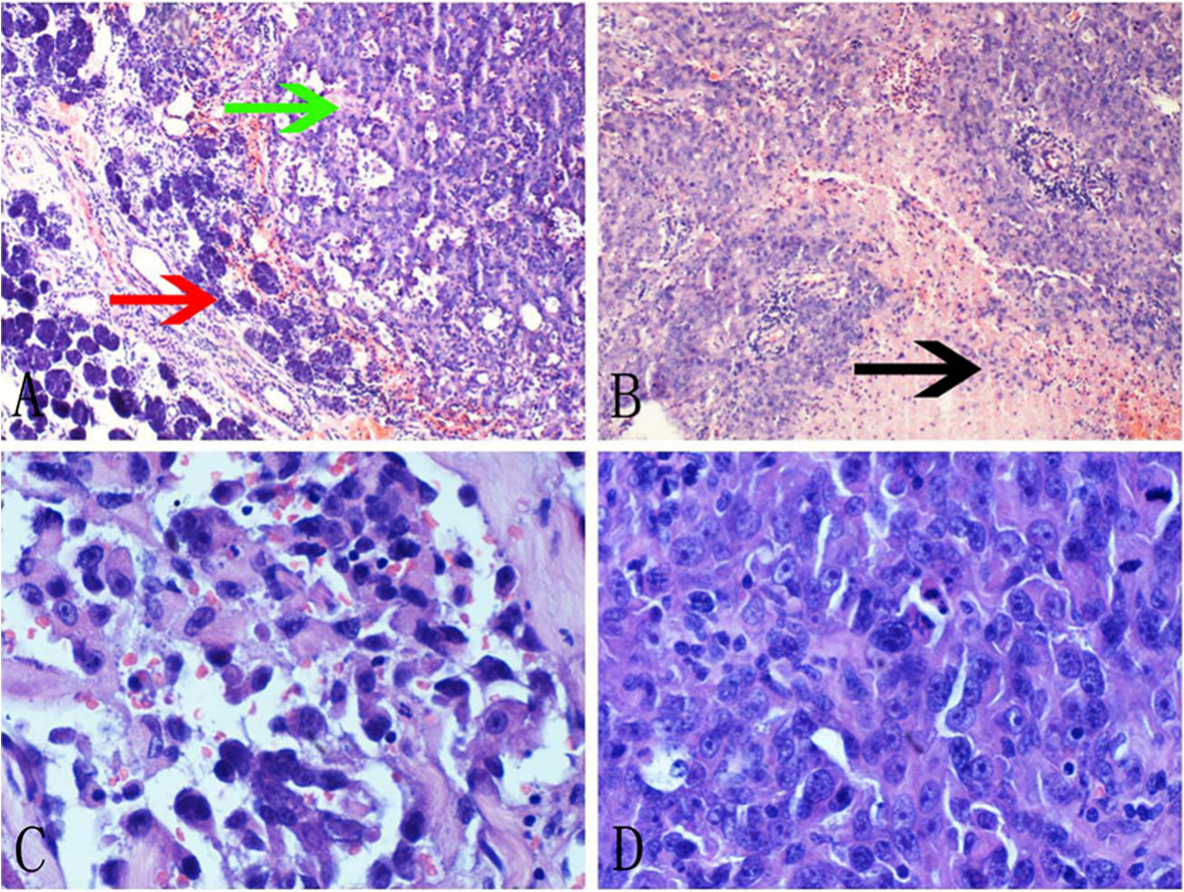
**A, The tumor cells were arranged in nests on the right area (green arrow); while the normal parotid gland tissue was on the left area (red arrow). B**, The area of necrosis and hemorrhage could also be seen easily (black arrow). **C**, The large vascular spaces could be seen on peripheral area of the whole section. **D**, Solid areas showed anaplastic cells with prominent nucleoli and no clear-cut vasoformation.

Grossly, the resected parotid gland tissue measured 3.2 × 2.8 × 1.6 cm with irregular shape, and was not well circumscribed, the cut surface showed grey-red or grey –white in colour with focal hemorrhage. Histologically, the tumor was made up of large round or polygonal epithelioid cells which were predominantly arranged in solid sheets or nests. The area of necrosis and hemorrhage could also be seen easily. There was no area of spindle cells in the whole section. The cells had pale to basophilic copious cytoplasm and vesicular nuclei, prominent nucleoli with significant anaplastic features. Focally, the tumor cells were arranged into gaping vessel-like spaces or sinusoid-like spaces (Figure 1).

**Figure 2:**
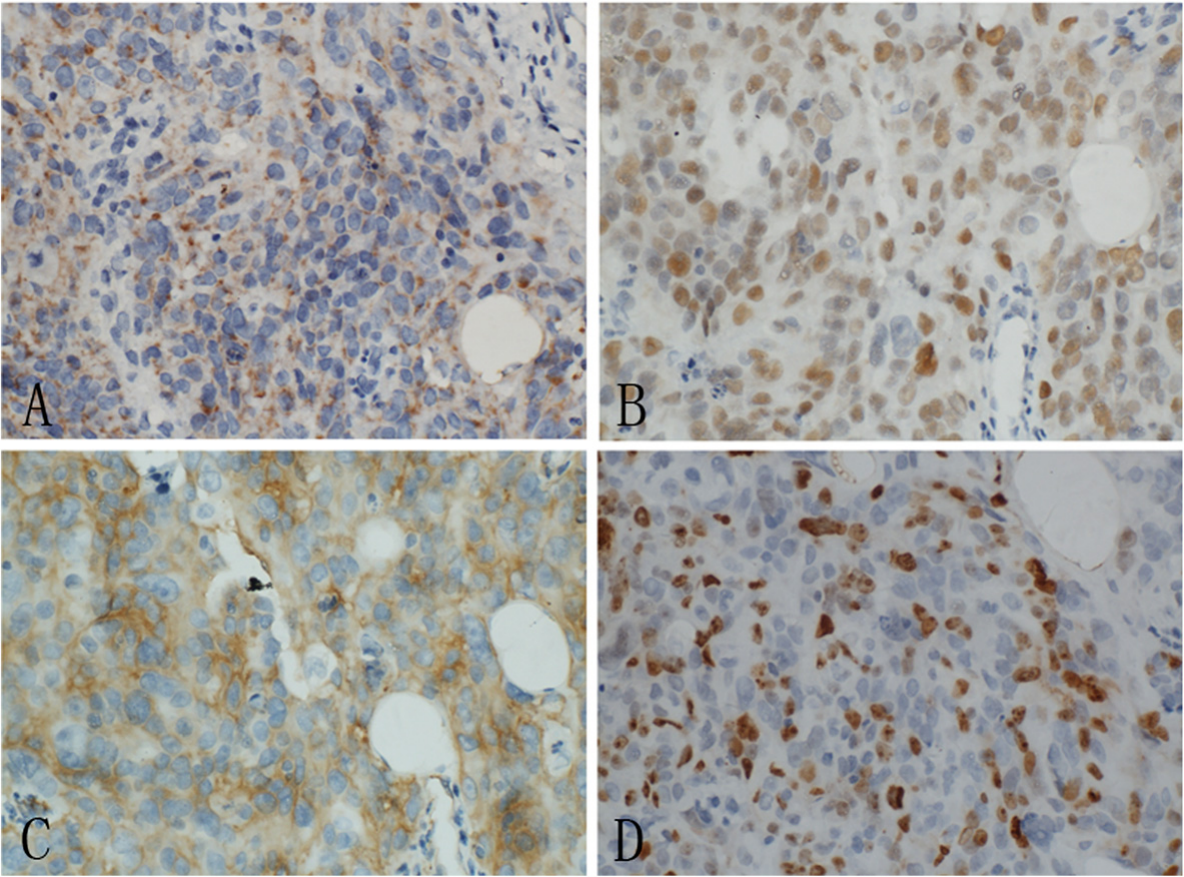
**A, Diffuse pankeratin staining highlighted the tumor cells. B**, The majority of the tumor cells was positive for p63. **C**, Diffuse and strong expression of CD31could be seen in the tumor cells. **D**, Almost 50% of tumor cells were positive for Ki67.

Immunohistochemistry showed the tumor cells were diffuse (over 50% of tumor cells) positive for pan-CK, p63 and Vimentin, CD31 was also strongly expressed in tumor cells. In addition, a focal and weak immunostaining was seen for CK18. The tumor cells were negative for CD34, CK 5/6, CK7, CK20, S100, desmin, α–SMA, CD45, CD30, CD68 and HMB45. Ki67 was expressed approximately in 50% of all tumor cells (Figure 2). According to the morphological and immunohistochemical findings, the tumor was diagnosed as an epithelioid angiosarcoma.

## Discussion

Angiosarcoma predominantly arises in the skin or superficial soft tissue. Less frequently, it can occur in various organs and has been reported in ovary [5], oral cavity [3], small intestine [6], thyroid [7], pleura [8], lung [9], testis [10,11] and parotid [2,3], even in fibroadenoma [12] and phyllodes tumour [13]. Occasionally, it also may be part of dedifferentiated liposarcoma [14] or mixed malignant tumors [15,16]. Epithelioid angiosarcoma most commonly involves deep soft tissue. Histologically, epithelioid angiosarcoma predominantly consists of sheets of highly atypical round cells with prominent nuclei, resembling poorly differentiated carcinoma, so, it may cause a great diagnostic confusion [4]. The diagnosis hint of angiosarcoma may be the existence of sinusoid-like spaces or the remarkable necrosis or hemorrhage. Unfortunately, there is a variant in squamous cell carcinoma, namely, pseudoangiosarcomatous cell carcinoma which also shows anastomosing vascular and gland-like spaces mimicking angiosarcoma [17]. So, in addition to histological findings, immunostaining must be used for distinguishing the two entities.

The endothelial cells show reactivity for several markers, including CD31, CD34 and von Willebrand factor (factor VIII). Among them, CD31 is considered the most sensitive and most specific endothelial cell marker. Epithelioid angiosarcoma is positive for CD31, but it is classically negative for CD34. In our case, there was strong and diffuse expression for CD31 in almost all tumor cells, and CD34 expression was absent. So we made the diagnosis of epithelioid angiosarcoma.

Some papers have documented that angiosarcoma or epithelioid angiosarcoma can express cytokeratin [18]. Miettinen M *et al.* found that epithelioid angiosarcomas were often positive for CK8 and CK18 (approximately 50%) [18]. Therefore, CK may be not helpful in distinguishing epithelioid angiosarcoma from squamous cell carcinoma. In our case, although CK showed a diffuse expression, but contrary to the diffuse and strong cytokeratin immunoreactivity in most of the carcinoma, the staining seemed to be relatively weak. This may represent an important clue to the possibility of non-epithelial lesions.

P63 is a p53 homolog that is expressed in various normal epithelial tissues and epithelial malignancies [19,20]. Many pathologists believed that immunohistochemical staining for p63 protein was a useful marker for distinguishing spindle cell carcinoma from sarcoma [21,22]. Jo VY *et al.* also demonstrated that p63 immunohistochemical staining is limited in soft tissue tumors, and failed to find expression of P63 in 20 cases of angiosarcomas [23]. On the contrary, in our case we found diffuse p63 expression in the tumor cells. So far, there is only one literature that described the positive expression of p63 in angiosarcoma, that is, Kallen ME *et al.* reported nuclear p63 expression might been seen in 20%–30% of malignant vascular tumors, including angiosarcoma, epithelioid angiosarcoma and epithelioid hemangioendothelioma [24]. Senoo M *et al.* found that p63 can affect expression of vascular endothelial growth factor via interactions with hypoxia inducible factor-1α [25]. Consequently, it is reasonable that p63 can express in vascular tumors, and p63 may not be as specific for the epithelial differentiation as the literatures reported [21-23]. Moreover, in contrast to described by Kallen ME *et al.* that only a minority of tumor cell nuclei were immunoreactive in most p63-positive vascular tumors [24], in our case over 75% of the tumor cells was positive. The p63 expression in angiosarcoma represents another diagnostic pitfall.

## Conclusion

Because of the rarity and closely mimicking carcinoma, epithelioid angiosarcoma is misdiagnosed easily, especially when one unfamiliar with this entity. CK and p63 are usually considered as the markers for carcinoma, but our result indicates the co-expression of them can be seen in angiosarcoma, this may be a further diagnostic pitfall. To avoid the misdiagnosis, using a panel of antibodies including endothelial cell markers is quite essential.

## Consent

Written informed consent was obtained from the patient for publication of this case report and accompanying images. A copy of the written consent is available for review by the Editor-in Chief of this Journal.

## Competing interests

The authors declare that they have no competing interests.

## Authors’ contributions

**L**XY participated in the histopathological evaluation, performed the literature review, acquired photomicrographs and drafted the manuscript. LY carried out the immunohistochemical stains evaluation. ZY and YJH conceived and designed the study. WEH gave the final histopathological diagnosis and revised the manuscript. All the authors read and approved the final manuscript.
